# Obstructive Sleep Apnea Treatment and the Evaluation of Clinical Effectiveness of Uniquely Designed Oral Appliance Therapy Device

**DOI:** 10.7759/cureus.59579

**Published:** 2024-05-03

**Authors:** Joseph Ojile, Matthew Uhles, Sabina Alisic, Kevin Postol, James Lillenberg

**Affiliations:** 1 Sleep Medicine, Clayton Sleep Institute, Saint Louis, USA; 2 Sleep Medicine and Colorectal Cancer, Clayton Sleep Institute, Saint Louis, USA; 3 Dental Surgery, Gateway Center for Sleep Apnea & TMJ Therapy, Saint Louis, USA; 4 Dental Surgery, Lillenberg Dental Sleep, Saint Louis, USA

**Keywords:** apnea-hypopnea index (ahi), effectiveness, compliance, mda: mean disease alleviation, osa: obstructive sleep apnea, oat: oral appliance therapy

## Abstract

Background: Continuous positive airway pressure (CPAP) has been considered first-line therapy for patients with obstructive sleep apnea (OSA); however, adherence to the therapy is suboptimal. Oral appliance therapy (OAT) is an alternative to CPAP that may lend to better patient adherence, quality of life, and overall patients’ effectiveness of therapy.

Methods: This was a prospective, single-site, non-randomized study to evaluate the clinical effectiveness of a uniquely designed OAT device with an embedded adherence tracking chip in the treatment of mild and moderate OSA patients over three months. The effectiveness of OAT therapy was defined as the numerical product of efficacy and adherence. The efficacy of the device was defined as the change from baseline in the apnea-hypopnea index (AHI). Adherence was based on usage for a minimum of 4 hours/night of use, for at least five out of seven nights a week.

Results: 45 participants fitted with the OAT device completed at least one follow-up visit and had recordable objective data. Average patient wearing time was 7 hours/night and a reduction of the AHI from 16.4 events/hour to 5.7 events/hour after three months of use. Mean disease alleviation (MDA), which serves as a measure of the overall therapeutic effectiveness, was 62% when looking at 4 hours/night of usage. As the comfort of the device is related to wearing time, subjective data indicated the optimum first-time fit of the device.

Conclusion: The study OAT device was well tolerated throughout the study. When both efficacy and adherence are considered, OAT can be a clinically effective tool to treat OSA.

## Introduction

Obstructive sleep apnea (OSA) is a common sleep disorder occurring in approximately 936 million people [1,2]. OSA is characterized by repetitive pharyngeal collapse during sleep, leading to sleep disruption, arousal, and/or oxyhemoglobin desaturation. Common symptoms of OSA include loud and chronic snoring, frequent awakenings during the night accompanied by choking or gasping for breath, daytime sleepiness, morning headaches, dry mouth or sore throat upon waking, difficulty concentrating, and irritability. Untreated OSA is associated with excessive daytime sleepiness, reduced cognitive function, hypertension, cardiovascular disease, stroke, reduced health-related quality of life (HR-QOL), increased mortality, and motor vehicle accidents due to sleepiness [3-15]. Patients are often prescribed continuous positive airway pressure (CPAP) therapy for the treatment of OSA. CPAP therapy is generally recommended for mild, moderate, and severe OSA. It is particularly efficacious for individuals with higher body mass index (BMI). Despite its efficacy in reducing OSA events, many patients struggle with consistent nightly use of CPAP. Studies have shown suboptimal adherence, with only about 50% using CPAP ≥4 hours per night after six months and as low as 17% using CPAP therapy at six years [16,17].

Non-adherence to CPAP therapy overall remains a tremendous roadblock to OSA therapy [18-20]. While CPAP continues to be a key non-invasive therapy for those who can tolerate it throughout the night, other non-invasive therapies must be considered with a patient-centered approach to effective OSA treatment.

The goal of this study is to evaluate the safety and effectiveness (efficacy and adherence) of a uniquely designed oral appliance device (on patients that were naïve to OSA therapy or were CPAP non-compliant) that supports nasal breathing and provides optimal OSA therapy to patients comfortably. In addition, QOL and satisfaction of patients were collected and analyzed.

## Materials and methods

Study design and participants

**Figure 1:**
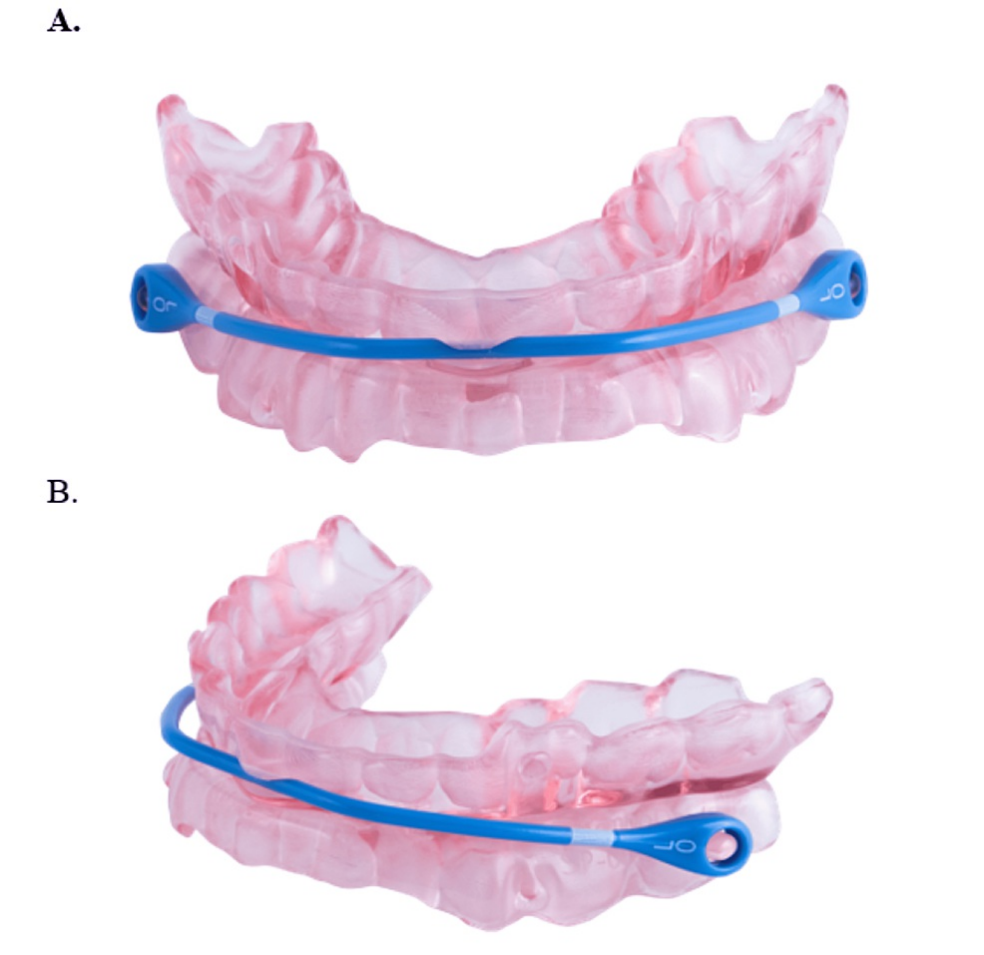
SomnoDent® Avant™ A, SomnoDent® Avant™ (front); B, SomnoDent® Avant™ (side) Permission was granted by SomnoMed Ltd, Sydney, Australia to use Figures A, SomnoDent® Avant™ (front), and B, SomnoDent® Avant™ (side).

The study was a prospective, single-site, non-randomized study, in the treatment of mild and moderate OSA patients in a clinical setting for both initial diagnosis (naïve to therapy) and those not adhering to their CPAP regimen. The study evaluated the clinical effectiveness of a uniquely designed oral appliance therapy (OAT) device (SomnoDent® Avant™ SomnoMed Ltd, Sydney, Australia) (Figure 1).

The OAT device is a designed titratable custom-made (with a passive mouth closing) device (SomnoDent® Avant™ SomnoMed Ltd, Sydney, Australia) that has a frontal exchangeable advancement strap of fixed lengths and its specific titration mechanism that supports lateral movement. The positioning of the strap on the anterior upper tray and the attachment to the posterior of the lower tray act to passively close the mouth, encouraging nasal breathing during sleep and after apnea events. The device also uses a patented material inner liner that cushions and supports the teeth for increased mouth comfort. The OAT device uses a unique strap design to allow lateral movement, limit mouth opening, and encourage nasal breathing while sleeping. Additional advancement adjustments to the device can occur at home by the patient after receiving the device from the dentist. These advancement adjustments are in 1 mm step increments for a maximal adjustment of up to 9 mm. 

During the study, the OAT device had an attached thermal adherence tracking chip (Dentitrac, BRAEBON Medical Corporation, Ontario, Canada) in order to gather objective adherence data. All participants provided consent prior to being enrolled. The study was approved by the Allendale Investigational Review Board (an independent/external ethics committee not affiliated with any of the institutions noted in this study) and was conducted in accordance with the Declaration of Helsinki.

Participants were screened either in person, by phone, or remotely to see if they were interested and met eligibility for the study. Those participants eligible and interested then signed an Institutional Review Board (IRB) approved consent. Inclusion criteria consisted of being 18 years of age or older with a diagnosis of OSA and AHI between 10 and 30 events per hour. The diagnostic and efficacy studies of AHIs (using a 3% hypopnea scoring criteria) were confirmed using a home sleep study (HST - Itamar WatchPAT One). Participants were excluded if they had a history of respiratory failure or insufficiency, suspected or known neuromuscular disease, moderate chronic obstructive pulmonary disease (COPD), or any condition with an elevation of arterial carbon dioxide levels while awake or the requirement for supplemental oxygen (at night or continuous) or mechanical ventilation. Participants also could not have undergone surgery of the upper airway, mouth, nose, sinus, within the previous 90 days, or surgery at any time for the treatment of OSA. Shift workers and participants with removable dentures or those with contraindications identified by the study dentist (periodontal disease, insufficient teeth for the device, or other medical reasons that would prevent wearing the device) were excluded from the study.

Study procedures

Participants meeting study criteria completed baseline questionnaires, including the Short Form-36 (SF-36) QoL, Epworth Sleepiness Scale (ESS), and the Functional Outcomes of Sleep Questionnaire (FOSQ). Participants had an intra-oral scan performed from which the oral appliance was constructed. Participants returned for dispensing, fitting, and titration of the study device. Patients were initially titrated to a maximal starting protrusion rate of 60% advancement of the lower jaw to make sure each patient was getting an efficacious treatment on the OAT device. Patients were then sent home and instructed to start wearing their OAT device for OSA treatment. Advancement adjustments (in 1 mm steps up to 9 mm advancement) to the OAT device could be made at home by the patient based on the patient’s level of comfort wearing the device. 

After starting OAT treatment, participants were instructed to document daily adherence in an electronic sleep diary and return to the clinic at approximately one month and three months. At these visits, participants had their vital signs recorded, an HST (Itamar WatchPAT One) performed (with the OAT device in the patient's mouth), and the thermal chip data collected to assess therapy adherence. Additionally, at the three-month visit, participants were asked to complete the following questionnaires: SF-36 QoL, ESS, and the FOSQ.

Endpoints and analyses

The effectiveness of OAT therapy for a participant is defined as the numerical product of efficacy and adherence [21]. The efficacy of the device is defined as the change from baseline in AHI, based on an HST. Adherence is based on usage data obtained from the thermal chip attached to the OAT study device, looking at usage for a minimum of four hours/night of use for at least five out of seven nights a week. The primary effectiveness analysis used an analysis of variance (ANOVA) model in the multiple imputation setting, with effectiveness at three months as the dependent variable and the study group as the explanatory variable. Mean estimates of effectiveness along with confidence intervals are provided for each study group. Questionnaire data is summarized descriptively.

## Results

The study initially enrolled 57 participants. Two participants did not receive a device, and four participants were either lost to follow-up, non-compliant with the protocol, or did not want to continue and were discontinued from the study leaving 53 participants who completed at least one follow-up visit. Baseline information for all participants and for those that had at least one follow-up visit are presented in Table 1.

**Table 1:** Baseline demographics and participant characteristics Data are n(%) or mean (SD). CPAP, continuous positive airway pressure; BMI, body mass index

Characteristic	Baseline (N=57)	1 month/3 months cohort (N=53)
Age (years)	51.5±11.7	52.2±11.5
Male	38 (66.7)	35 (66.0)
Female	19 (33.3)	18 (34.0)
BMI	31.5±7.3	31.2±7.3
History of snoring	54 (94.7)	50 (94.3)
Naïve patients	29	28
CPAP non-compliant patients	28	25

During the study, there were instances of thermal chip shortages, malfunctions, and non-activations that caused seven participants to have unrecoverable data. Usage for the remaining 45 participants averaged 6.8 hours/day (Table 2). Change in AHI over time was statistically significant, going from 16.3 events/hour at baseline down to 8.0 events/hour after one month of wear and 5.7 events/hour after three months (p<0.0001) (Table 3). Mean disease alleviation (MDA), calculated by adherence to usage of the OAT for a minimum of four hours/night of use, and AHI changes from baseline to three months was 62% (Table 4). Statistically significant improvement was measured on both the ESS and FOSQ sleep questionnaires at three months when compared to baseline (Table 5) and a significant difference in the SF-36 QoL vitality domain (Table 6). All adverse events related to normal use of OAT devices were manageable with the standard dentist follow-up or resolved on their own. There were no reported device deficiencies.

**Table 2:** Device usage baseline through three months

Device usage	3 months (average hours/day)
N	45
Mean±SD	6.8±1.4
Median	7.0
Min, max	2.9, 9.8
95% CI	6.4, 7.2

**Table 3:** Sleep questionnaire results (FOSQ and ESS) FOSQ score range: 5-20 points, with higher scores indicating better functional status. ESS score range: 0-24, with higher scores indicating greater daytime sleepiness. Scores greater than or equal to 11 are generally considered to be abnormal (positive) for excessive daytime sleepiness [22]. FOSQ, Functional Outcomes of Sleep Questionnaire; ESS, Epworth Sleepiness Scale

Category	Baseline	3 months	3-month change (paired)
FOSQ score			
N	53	51	51
Mean ± SD	16.1±2.6	17.6±2.5	1.5±2.2
Median	16.7	18.3	1.3
Min, max	10.0, 20.0	8.8, 20.0	-3.5, 6.3
95% CI	15.4, 16.8	16.9, 18.3	0.9,2.1
p-value			<0.0001
Improved			70.6% (36/51)
ESS score			
N	53	51	51
Mean ± SD	8.6±4.7	5.5±3.6	-3.0±4.1
Median	8.0	4.0	-3.0
Min, max	0.0, 19.0	0.0, 19.0	-14.0, 6.0
95% CI	7.3, 9.9	4.5, 6.5	-4.2, 1.8
p-value			<0.0001
Improved			68.6% (35/51)

**Table 4:** Paired overscored AHI results (n=49 paired datasets) The p-value is a paired t-test comparing the difference from baseline. AHI, apnea-hypopnea index

Category	Baseline	1 month	1-month change from baseline	3 months	3-month change from baseline
N	49	49	49	49	49
Mean ± SD	16.3±7.2	8.3±5.8	-8.0±7.2	5.7±3.9	-10.6±7.0
Median	14.5	7.5	-7.6	4.4	-9.3
Min, max	6.8, 42.3	0.5, 30.1	-31.0, 8.8	0.4, 14.8	-30.1, 1.8
95% CI	14.2, 18.4	6.6, 10.0	-10.1, -5.9	4.6, 6.8	-12.6, -8.6
p-value			0.0000		0.0000
Improved			89.8% (44/49)		98.0% (48/49)

**Table 5:** MDA ^a^AHI alleviated at three month=mean three-month change/mean bassline. ^b^MDA=objective adherence×therapeutic efficacy. MDA, mean disease alleviation; AHI, apnea-hypopnea index

Category	3-month paired adherence, n/N (%)	AHI 3-month % alleviated^a^	MDA^b^
Adherence of ≥ 4 hours	43/45 (95.6)	-10.6/16.3 = 0.650	62%

**Table 6:** SF-36 domains summary (p-value is paired t-test comparing for the difference from baseline) SF-36, Short Form-36

Category	Baseline	3 months	3-month change from baseline
Physical functioning			
N	50	50	50
Mean ± SD	80.5±21.6	84.5±18.4	4.0±17.7
Median	90.0	90.0	0.0
Min, max	25.0, 100.0	25.0, 100.0	-50.0, 75.0
95% CI	74.4, 86.6	79.3, 89.7	-1.0, 9.0
p-value			0.1165
Improved			40.0% (20/50)
Role physical			
N	50	50	50
Mean ± SD	69.4±36.3	77.0±35.7	7.6±28.6
Median	75.0	100.0	0.0
Min, max	0.0, 100.0	0.0, 100.0	-75.0, 100.0
95% CI	59.1, 79.7	66.9, 87.1	-0.5, 15.7
p-value			0.0662
Improved			30.0% (15/50)
Role emotional			
N	50	50	50
Mean ± SD	82.0±32.5	84.0±29.6	2.0±35.9
Median	100.0	100.0	0.0
Min, max	0.0, 100.0	0.0, 100.0	-100.0, 100.0
95% CI	72.8, 91.2	75.6, 92.4	-8.2, 12.2
p-value			0.6953
Improved			18.0% (9/50)
Vitality			
N	50	50	50
Mean ± SD	44.9±20.8	58.9±20.0	14.0±21.4
Median	40.0	60.0	15.0
Min, max	0.0, 90.0	5.0, 100.0	-30.0, 60.0
95% CI	39.0, 50.8	53.2, 64.6	7.9, 20.1
p-value			0.0000
Improved			68.0% (34/50)
Role emotional			
N	50	50	50
Mean ± SD	77.1±16.5	77.8±14.7	0.6±14.5
Median	80.0	80.0	0.0
Min, max	28.0, 100.0	32.0, 96.0	-40.0, 32.0
95% CI	72.4, 81.8	73.6, 82.0	-3.5, 4.7
p-value			0.7711
Improved			44.0% (22/50)
Social functioning			
N	50	50	50
Mean ± SD	82.4±18.2	83.1±22.9	0.8±25.3
Median	88.0	88.0	0.0
Min, max	38.0, 100.0	0.0, 100.0	-87.0, 50.0
95% CI	77.2, 87.6	76.6, 89.6	-6.4, 8.0
p-value			0.8240
Improved			38.0% (19/50)
Bodily pain			
N	50	50	50
Mean ± SD	72.6±24.5	70.3±24.3	-2.3±28.7
Median	78.0	79.0	0.0
Min, max	13.0, 100.0	0.0, 100.0	-87.0, 57.0
95% CI	65.6, 79.6	63.4,77.2	-10.5, 5.9
p-value			0.5735
Improved			44.0% (22/50)
General health			
N	50	50	50
Mean ± SD	64.6±17.1	66.5±16.9	1.9±15.9
Median	65.0	70.0	0.0
Min, max	30.0, 95.0	30.0, 90.0	-40.0, 40.0
95% CI	59.7, 69.5	61.7, 71.3	-2.6, 6.4
p-value			0.4022
Improved			42.0% (21/50)

## Discussion

The AHI is a diagnostic tool for determining the presence and severity of OSA, which uses and combines the average number of apneas and hypopneas that occur per hour of sleep. AHI is categorized into mild (five to 15 events/hour), moderate (15-30 events/hour), and severe (>30 events/hour) [23]. In this study, patients' mean AHI went from the moderate range (16.3±7.2) to mild (5.7±3.9) after three months. Although AHI is considered the gold standard metric for OSA severity, it does not take into account all other adverse outcomes that affect patients, and it does not take into account the total effectiveness of therapy [24].

The clinical effectiveness of any therapy for OSA needs to consider adherence to the prescribed treatment. Vanderveken et al. developed an objective effectiveness measure for OSA therapies [25]. The metric “mean disease alleviation” provides an assessment of treatment effectiveness by considering not only the reduction in AHI but also the patient adhering to their therapy on a nightly basis. The MDA provides an opportunity to compare various OSA therapies in the effectiveness of patient’s treatments. The MDA achieved in this study of 62% effectiveness is comparable with the MDA for CPAP of 60% described in the study by Every JD et al. [26].

Treatment of OSA is essential for improvement in patients’ health. The gold standard for OSA therapy is CPAP for the majority of patients. CPAP is highly efficacious for OSA treatment when used as directed (Centers for Medicare & Medicaid Services (CMS) requires a full 90-day (or three-month period) to wear CPAP for a minimum of four hours/night of usage for 70% of the time) [27]. However, overall adherence to CPAP continues to remain suboptimal because of various factors such as mask leaks, claustrophobia, difficulty exhaling air, device-related issues, etc., leading to lower overall effectiveness of this treatment method [28,29]. 

The popularity of non-invasive OSA therapies, like OAT, such as the one featured in this study, continues to grow in popularity. These alternative (to CPAP) treatments are gaining traction due to their ease of use and increased comfort, leading to greater adherence by patients and potentially enhanced effectiveness. Oral appliances, that are custom-designed to fit each patient’s oral structure, play a significant role in treating OSA [30]. In the current study, the dentists had the opportunity to complete a survey (on 52 of the 53 patients) following their fitting experience with each patient. The dentists reported that over 90% (47/52) of patients had an “excellent” or “good” fit on the patient’s teeth at the initial fitting, with the remaining only needing some minor adjusting to fit properly. A properly fitted custom-made oral appliance can enhance comfort but also increase the probability of a patient maintaining high adherence to therapy [30].

Untreated/undertreated OSA can cause excessive daytime sleepiness and reduced cognitive function and QoL [30]. When comparing CPAP to OAT, we are learning that OAT is non-inferior to CPAP in improving certain health outcomes such as hypertension, disease-specific QoL, and neurobehavioral (daytime sleepiness) improvements, while at the same time providing higher adherence [31-33]. In the present study, data demonstrated a statistically significant improvement in QoL and reduced daytime sleepiness after three months (Table 5) of OAT use, similar to prior studies performed on OAT [33].

When selecting therapy (CPAP or OAT) for the management of OSA, it is crucial to adopt a tailored approach and not a one-size-fits-all approach. This involves considering updated evidence, treatment feasibility, and individual patient characteristics. Carefully assessing these factors, healthcare providers can tailor treatment plans to best meet each patient’s needs for optimizing treatment therapy in managing OSA. 

Study limitations

While this study provides insights into the newly designed OAT device, it should be noted that there are limitations, particularly regarding the generalizability of the results. The study was not a head-to-head (i.e., OAT vs CPAP) randomized comparison clinical trial to analyze by the intention-to-treat (ITT) approach. This may introduce biases and affect the generalizability of our findings. However, despite this limitation, our study provides valuable insights into the study device's ability to treat OSA. Future research should consider a more rigorous randomized controlled designed trial to more accurately assess the comparative effectiveness of OAT and CPAP therapies for OSA treatment. Study patients who dropped out of this study were individually evaluated by the site to explore alternative clinic options for treating their OSA. 

Because this was a single-site study and not a multi-site trial, a potential limitation would be the homogeneity of the patient population in this study. The absence of representation from a more diverse patient population (with an increased number of patients) in the United States could impact the quality of the findings. Future research incorporating a more diverse sample could significantly enhance our understanding of the effectiveness of the OAT device across various populations. By exploring the anthropometric and metabolic characteristics of those participants, researchers can gain valuable insights into how individual differences may impact therapy treatment outcomes. Understanding the valuable insights gathered from this three-month study was key to understanding the therapeutic effectiveness of patients in treating OSA. Monitoring patients' therapy treatment over extended periods, such as one, three, and five years, will be essential for assessing the study OAT device's continuous efficacy (including continuous device advancement adjustments made by patients at home) and adherence. Longitudinal studies can offer more insights into the long-term effects of treatment and provide a more comprehensive understanding of the OAT device’s performance and benefits over time in treating patients with OSA. 

## Conclusions

The study provides valuable insights into the benefits of using the titratable, custom-made OAT study device for patients diagnosed with mild to moderate OSA. Throughout the study, the device was well tolerated, with the average patient wearing it for approximately seven hours of the night. Additionally, a significant reduction in the AHI was seen, decreasing from 16.4 events/hour to 5.7 events/hour after three months of use. The MDA, which considers both efficacy and adherence, revealed a 62% treatment success rate, indicating a high level of treatment effectiveness. When both efficacy and adherence are considered, the OAT study device can be a clinically effective first-line treatment tool to treat mild and moderate OSA while improving the overall QoL of patients.
